# The Anxiety-Buffer Hypothesis in the Time of COVID-19: When Self-Esteem Protects From the Impact of Loneliness and Fear on Anxiety and Depression

**DOI:** 10.3389/fpsyg.2020.02177

**Published:** 2020-11-10

**Authors:** Alessandro Rossi, Anna Panzeri, Giada Pietrabissa, Gian Mauro Manzoni, Gianluca Castelnuovo, Stefania Mannarini

**Affiliations:** ^1^Section of Applied Psychology, Department of Philosophy, Sociology, Education, and Applied Psychology, University of Padua, Padua, Italy; ^2^Interdepartmental Center for Family Research, University of Padua, Padua, Italy; ^3^Unit of Psychology and Neuropsychology, Maugeri Scientific Institutes IRCCS, Novara, Italy; ^4^Psychology Research Laboratory, Ospedale San Giuseppe, IRCCS, Istituto Auxologico Italiano, Verbania, Italy; ^5^Department of Psychology, Catholic University of Milan, Milan, Italy; ^6^Department of Psychology, eCampus University, Novedrate, Italy

**Keywords:** COVID-19, anxiety buffer hypothesis, terror management theory, anxiety, depression, self-esteem, fear, loneliness

## Abstract

**Introduction:**

The coronavirus (COVID-19) disease has spread worldwide, generating intense fear of infection and death that may lead to enduring anxiety. At the same time, quarantine and physical isolation can intensify feelings of dispositional loneliness that, by focusing on thoughts of disconnection from others, can trigger intense anxiety. Anxiety, generated by both fear of COVID-19 and dispositional loneliness, can activate negative expectations and thoughts of death, potentially generating alarming depressive symptoms. However, the *anxiety-buffer hypothesis* suggests that self-esteem acts as a shield (buffer) against mental health threats – fear and loneliness – thus hampering anxiety and depressive symptoms.

**Objective:**

This study aims to test the process – triggered by COVID-19 fear and loneliness – in which self-esteem should buffer the path leading to anxiety symptoms, then to depression.

**Methods:**

An observational research design with structural equation models was used. A sample of 1200 participants enrolled from the general population answered an online survey comprising: the fear of COVID-19 scale, the UCLA loneliness scale, the Rosenberg self-esteem scale, and the anxiety and depression scales of the Symptom Checklist-90-Revised.

**Results:**

Structural equation models showed the link between anxiety symptoms (*mediator*) with both the fear of COVID-19 and dispositional loneliness (*predictors*), as well as its association with consequent depressive symptomatology (*outcome*). In line with the anxiety-buffer hypothesis, self-esteem mediated the relationship between the predictors and their adverse psychological consequences.

**Conclusion:**

Self-esteem represents a protective factor from the antecedents of depression. Targeted psychological interventions should be implemented to minimize the psychological burden of the disease whilst promoting adaptation and positive psychological health outcomes.

## Introduction

The novel coronavirus (COVID-19) is a new severe and potentially mortal disease threatening to infect the entire human population given that there is no prior immunity and not even a well-established cure or vaccine yet (Baud et al., 2020).

COVID-19 displays a variety of clinical features ranging from asymptomatic presentations (20–50%), fever (>90%), cough (75%), shortness of breath (50%), up to acute respiratory distress syndrome, and death (Byambasuren et al., 2020; Center for Disease Control and Prevention, 2020; Jiang et al., 2020). Categories of people at higher risk of developing severe complications of COVID-19 are older adults and people with previous underlying medical conditions, such as hypertension, cardiovascular disease, respiratory disease, and cancer (Liu et al., 2020; Armitage and Nellums, 2020; Zheng et al., 2020). The contagion occurs from an infected person, even without obvious symptom manifestation, via respiratory droplets that can be inhaled or can land on surfaces which are later in contact with other people.

Due to its high transmissibility, since December 2019 COVID-19 has been rapidly spreading worldwide causing the current pandemic (World Health Organization [WHO], 2020). Across the world, strict preventive policies were adopted to contain the outbreak of COVID-19 – including social distancing and social isolation. Nevertheless, the magnitude of this pandemic has generated serious concerns about its social and economic consequences both in the short and long-term (Cerami et al., 2020). Thus, COVID-19 represents an epochal economic, physical, and biological threat to everyone’s lives.

Therefore, beyond threatening people’s physical conditions, COVID-19 is accompanied by remarkable psychological burdens heavily affecting people’s mental health (Brooks et al., 2020; Torales et al., 2020; Wang et al., 2020). Similar to other physical diseases, COVID-19 represents a specific dangerous trigger activating a *“fight or flight”* reaction of (functional) fear focused on illness and death (Schaller et al., 2015; Harper et al., 2020). The COVID-19 pandemic-related fear also led to counterproductive and detrimental behaviors for the whole society (i.e., demanding unnecessary medical care, excessively protecting against the virus, and overstocking certain supplies) (Lin, 2020).

Moreover, fear of illness and death commonly lead to chronic vigilance for potential threats, thus contributing to the development of anxiety (i.e., the anticipation of a feared threat without a stimulus) that is future-oriented, unfocused, diffused, and extended to non-threatening situations (Barlow, 2002; Harding et al., 2008).

In turn, anxiety might trigger and catalyze depressive symptoms via the activation of processes including persistent preoccupations, negative expectations, thoughts about death (of themselves or significant others), and pervasive pessimism (Thompson et al., 2005; Starr and Davila, 2012). Depressive symptoms include feelings of sadness and loss, a negative view of the self, of the world, and of the future, thought and behavior are slowed down, and positive emotions are absent (Beck, 1979). Noteworthy, depressive symptoms spread widely during the COVID-19 pandemic, representing an alarming predictor of suicide-behaviors (McIntyre and Lee, 2020; Thakur and Jain, 2020).

At the same time, quarantine and physical distancing generated widespread feelings of isolation and loneliness – despite that fact that human connections were facilitated and granted by the use of communication technology (Russell, 1996; Usher et al., 2020). Indeed, the dispositional trait of loneliness may have a crucial role in perceiving and amplifying feelings of isolation, thus exacerbating the adverse psychological impact of the outbreak (Boffo et al., 2012). Indeed, dispositional loneliness is characterized by perceived disconnection from others and unpleasant feelings of isolation. Dispositional loneliness activates distressing thinking processes focusing on comparisons between the actual and the desired socio-relational situation. This contributes to the increase of unpleasant feelings and leads to the development of symptoms of anxiety that – in turn – lead to depressive symptomatology (Cacioppo et al., 2006, 2014; Santini et al., 2020). In other words, by activating (maladaptive) mechanisms and by influencing the brain and behavior, loneliness makes people more susceptible to the onset of anxious and depressive symptoms – thus representing an important risk factor for poor mental health (Fiese et al., 2002; Heinrich and Gullone, 2006; Hossain et al., 2020; Lunn et al., 2020; Zhou et al., 2020), long-term morbidity (i.e., cardiovascular), and mortality (Cacioppo et al., 2014; Leigh-Hunt et al., 2017; Rico-Uribe et al., 2018).

Consequently, both a fear of COVID-19 and dispositional loneliness could be considered as predictors of severe psychological symptoms of anxiety and depression, potentially leading to dismal effects, including extreme life-threatening behaviors (Santini et al., 2020; Thakur and Jain, 2020).

However, self-esteem – that is the individuals’ attitudes, beliefs, and evaluations toward the self – may buffer these adverse patterns. According to Becker (1971, 1973), self-esteem is built on deep-rooted personal values derived from a given social, relational, and cultural context, and it is reinforced by social validation and the feeling of being a valuable human being with a meaningful role in society given by meeting the standards of a given culture and worldview (Pyszczynski et al., 2004). More recently, the terror management theory (TMT) (Greenberg et al., 1986) postulated that individuals’ awareness of mortality – in this case elicited by COVID-19 – conflicts with the human intrinsic desire for life and tendency to survive, thus generating terrifying fears of death and then anxiety. In this framework, the anxiety-buffer hypothesis (ABH; Greenberg et al., 1992) theorizes that, by reconnecting the individual with an enlarged universe of meanings and values, self-esteem could act as a protecting shield (buffer) against the detrimental psychological effects of life-threats and stressors.

### Aims and Hypotheses

Considering this background, the present study aimed at testing the anxiety-buffer hypothesis during the COVID-19 pandemic. More in detail, self-esteem should buffer the relationships from both a fear of COVID-19 and dispositional loneliness to anxiety symptoms – that in turn lead to depressive symptoms. Moreover, specific hypotheses about each path (relationship) between variables were formulated:

H1: fear of COVID-19 and dispositional loneliness are positively associated with depressive symptomatology;
H2a: fear of COVID-19 predicts depressive symptomatology through anxiety symptoms (simple mediation) – without considering the buffering effect of self-esteem;
H2b: dispositional loneliness predicts depressive symptomatology through anxiety (simple mediation) – without considering the buffering effect of self-esteem;
H3: fear of COVID-19 and dispositional loneliness predict depressive symptomatology through anxiety symptoms (mediation) – without considering the buffering effect of self-esteem;
H4: fear of COVID-19 and dispositional loneliness predict depressive symptoms through self-esteem (buffering effect) and anxiety symptoms (multiple mediation).

In other words, it was hypothesized that a fear of COVID-19 and loneliness are associated with depressive symptomatology, but this relationship should be mediated by both anxiety and self-esteem. In particular, self-esteem should play a buffering role.

## Materials and Methods

### Procedure

An online survey was developed and disseminated using the Qualtrics software for data collection.

Firstly, the survey was administered to 20 participants – not included into analysis (A) to ensure whether the items were understandable by the general population and (B) to estimate an acceptable time for its fulfillment (8’–20’), so as to deal adequately with potentially biased responses: too fast – random answers – or too slow –in which the subject could have been interrupted during the completion.

Then, the snowball sampling method (Fricker, 2008) was used to recruit participants from the general population through personal invitations or materials advertised via social media platforms (i.e., Facebook, Twitter).

The recruitment materials provided details of what was required for participation in the study and a weblink to access the online questionnaire. The weblink directed potential participants first to further information on the research project in order to make an informed decision about study participation. Participants were informed that their responses were anonymous as well as that no economic payment was offered for their voluntary participation. Those who provided their consent online proceeded to the online questionnaire.

Inclusion criteria for the participants into the study were: (A) being a native Italian speaker; (B) being over 18 years old; and (C) providing informed consent. We excluded participants from the study who: (D) did not answer all the questions in the survey and (E) spent less than 8 min or more than 20 min completing the survey.

Data were collected in their entirety in a single week interval during the Italian quarantine to avoid confounding effects due to pandemic fluctuations. The study was approved by the Ethic Committee of the University of Padua in accordance with the Ethical standards of the Declaration of Helsinki.

### Sample Size Determination

Considering the statistical analyses used in this study (see designated section), the sample size was calculated *a priori* according to the “*n:q* criterion”: where *n* is the number of participants and *q* is the number of (free) model parameters to be estimated (Hu and Bentler, 1999; Muthén and Asparouhov, 2002; Yu, 2002). Consequently, ten subject per free parameter (10:73; *n*_*minimum*_ = 730) were guaranteed (Bentler and Chou, 1987; Marsh et al., 1988; Hu and Bentler, 1999; Boomsma and Hoogland, 2001; Muthén and Asparouhov, 2002; Yu, 2002; Flora and Curran, 2004; Tomarken and Waller, 2005).

### Participants

According to the inclusion criteria, 62 respondents were excluded from the study due to incomplete surveys (*n* = 35) and inappropriate completion times (*n* = 27).

The final sample was composed by 1200 participants [217 males (23.3%) and 713 females (76.7%), aged from 18 to 81 years (*mean* = 39.59, *SD* = 12.334)], the average time competing the survey was 11’0.27” (SD = 3’0.02”). A total of 965 respondents were from Northern Italy (80.4%), 165 were from central Italy (13.8%), and 70 participants were from Southern Italy and the islands (5.8%). Descriptive statistics of this sample are reported in Table 1.

**TABLE 1:** Socio-demographic characteristics of the sample.

	Mean	SD
**Age**	39.33	12.283
Number of persons living with	2.63	1.791

	**Frequency**	**Percentage**

**Sex**		
Male	217	18.1%
Female	983	81.9%
**Civil Status**		
Single	237	19.8%
In a relationship	379	31.6%
Married	484	40.3%
Divorced	86	7.2%
Widowed	14	1.2%
**Education**		
Elementary school	3	0.3%
Middle school	117	9.8%
High school	491	40.9%
Bachelor degree	462	38.5%
Master degree/Ph.D.	127	10.6%
**Work position at the time of the survey**		
Smart-working/smart studying	409	34.1%
Paid leave	38	3.2%
Time off work	17	1.4%
Compulsory leave	63	5.3%
Laid off	144	12.0%
Closure of the activity	100	8.3%
Still working at the workplace	205	17.1%
Unemployed	164	13.7%
Retired	60	5.0%
**Respondent – positive COVID-19 diagnosis**		
Yes (given the swab)	4	0.3%
No (given the swab)	139	11.6%
Unknown (not given the swab)	1057	88.1%
**Significant other – positive COVID-19 diagnosis**		
Yes (given the swab)	136	11.3%
No (given the swab)	166	13.8%
Unknown (not given the swab)	898	74.8%

### Measures

Socio-demographic information included sex, age, education, employment, Italian region of residence, number of persons living with, and confirmed positive COVID-19 diagnosis of the respondent and of his/her significant others. Table 1 reports the sample characteristics.

In addition, the following self-report measures were administered.

#### Fear of COVID-19 Scale – (FCV-19S)

The FCV-19S (Ahorsu et al., 2020; Soraci et al., 2020) is a 7-item self-report questionnaire aimed at assessing emotional, cognitive, physiological, and behavioral manifestations of COVID-19-related fear in the general population. Respondents are asked to indicate their degree of agreement to each statement on a 5-point Likert-type scale (ranging from 1 = *“strongly disagree”* to 5 = *“strongly agree”*) that provides a single-factor structure. Higher values indicate greater fear of COVID-19. In this study, the FCV-19S showed a high internal consistency (Cronbach’s alpha = 0.881).

#### University of California, Los Angeles, Loneliness Scale-Version 3 (UCLA-LS3)

The UCLA-LS3 (Russell, 1996; Boffo et al., 2012) is a 20-item self-report scale that evaluates the individuals’ global and prolonged (dispositional) perceived sense of loneliness through three dimensions: (A) sense “habitual” isolation, (B) perception of being socially isolated, and (C) “traits” and dispositional factors of loneliness (Boffo et al., 2012). In addition, a general dimension of “dispositional” loneliness is assumed. Respondents are asked to rate how often they feel the way described by each sentence on a 4-point Likert-type scale (ranging from 1 = *“never”* to 4 = *“always”*). Higher values indicate the presence of a greater feeling of loneliness. In this study, the UCLA-LS3 showed a high internal consistency for each dimension (A – Isolation: Cronbach’s alpha = 0.805; B – Relational connectedness: Cronbach’s alpha = 0.822; C – Trait loneliness: Cronbach’s alpha = 0.869) and for the general dimension (Cronbach’s alpha = 0.913).

#### Rosenberg Self-Esteem Scale (RSE)

The RSE (Rosenberg, 1965; Prezza et al., 1997) is one the most widely used self-report scales assessing global self-esteem in both clinical settings and in the general population. It consists of 10 positively and negatively worded statements evaluating feelings about one’s self. Respondents are asked to express their degree of agreement to each statement on a 4-point Likert-type scale (ranging from 1 = *“not at all”* to 4 = *“always”*), and it provides a single-factor structure. Higher values indicate a greater sense of global self-esteem. In the present sample, the RSE showed a high internal consistency (Cronbach’s alpha = 0.869).

#### Anxiety Subscale of the Symptom Checklist-90 Revised (SCL-90R – ANX)

The SCL-90R ANX subscale (Derogatis and Unger, 2010) is a 10-item self-report tool evaluating psychological, cognitive, and physical manifestations of anxiety during the previous week. For each statement, respondents are asked to rate the severity of their symptoms on a 5-point Likert-type scale (ranging from 1 = *“not at all”* to 5 = *“always”*). The ANX subscale provides a single factor structure. Higher values indicate a greater anxiety symptomatology. In this study, the ANX subscale showed a high internal consistency (Cronbach’s alpha = 0.932).

#### Depression Subscale of the Symptom Checklist-90 Revised (SCL-90R – DEP)

The SCL-90R DEP scale of Derogatis and Unger (2010) is a 13-item self-report tool evaluating emotive, cognitive, and somatic manifestations of depression during the previous week. Respondents are asked to rate the severity of their symptoms on a 5-point Likert-type scale (ranging from 1 = *“not at all”* to 5 = *“always”*). Also the DEP subscale provides a single factor structure. Higher values indicate a greater depressive symptomatology. In the present sample, the DEP subscale showed a high internal consistency (Cronbach’s alpha = 0.907).

### Statistical Analyses

All analyses were performed with the R statistical software system (v. 3.5.3) [R-core project (R Core Team, 2014, 2017)]. The following packages were used: psych (v. 1.8.12; Revelle, 2018), lavaan (v. 0.6-6; Rosseel, 2012; Rosseel et al., 2015), and semTools (v. 0.5-2; Jorgensen et al., 2019). Graphical representations were performed with graphViz in DiagrammeR (v.1.0.6.1; Iannone, 2018).

Preliminarily, a multivariate multiple regression analysis was performed to exclude the potential confounding effects of the following variables (covariates) on the aforementioned psychological constructs: (A) Italian region where respondents lived – as COVID-19 played out differently in Italy, (B) number of persons respondents lived with, (C) confirmed positive COVID-19 diagnosis of the respondents, and (D) confirmed positive COVID-19 diagnosis of the respondents’ significant other. Thus, external variables were simultaneously regressed on all the psychological constructs.

A Pearson correlation coefficient (*r*) was computed to evaluate the relationships between variables (Tabachnick and Fidell, 2014).

A structural equation modeling (SEM) approach with latent variables was followed (McDonald and Ho, 2002; Frazier et al., 2004; Weston and Gore, 2006; Iacobucci, 2008; Wiedermann and von Eye, 2015). A two-related separated predictors with a sequential multiple mediation model was specified (MacKinnon, 2012; VanderWeele and Vansteelandt, 2014; Daniel et al., 2015; Hayes, 2017). The following procedure was performed.

#### Step 1

Before examining the hypothesized model, the structural validity of each scale used in this study was tested by means of confirmatory factor analysis (CFAs). Considering the response scale of each of the questions administered in the study, the diagonally weighted least square (DWLS) estimator was used to perform each CFA separately (Hoyle, 2012; Brown, 2015; Kline, 2016; Lionetti et al., 2016). Model fit was assessed by means of the following fit indices – and recommended cutoff values (Bollen, 1989; Yu, 2002; Iacobucci, 2009; Hoyle, 2012; van de Schoot et al., 2012; Kline, 2016): (A) the Chi-square statistics (χ^2^), preferably non-statistically significant (*p* > 0.05) (Bentler and Bonett, 1980; Muthén and Muthén, 1998-2017; Barrett, 2007); (B) the root-mean-square error of approximation (RMSEA), with values below 0.08 indicating an “acceptable” model fit and values below 0.05 indicating a “good” model fit (Steiger and Lind, 1980; Steiger, 1990; Hu and Bentler, 1999; Barrett, 2007; van de Schoot et al., 2012); (C) the comparative fit index (CFI), with values between 0.90 and 0.95 for an “acceptable” fit (Browne and Cudeck, 1989; Bentler, 1990; van de Schoot et al., 2012; Brown, 2015) and higher than 0.95 to indicate a “good” fit (Bentler, 1990; Browne and Cudeck, 1992; Hu and Bentler, 1999), and (D) the standard root mean square residual (SRMR), with values lower than 0.08 considered a good model fit (Hu and Bentler, 1999; Hoyle, 2012).

#### Step 2

The Harman’s single-factor test was performed to check the potential “common method bias” (Harman, 1976; Podsakoff et al., 2003; Brown, 2015). Firstly, a correlated factors model was specified: according to the measurement model, seven correlated factors (FCV19 – single factor, UCLA-LS3– three factors, RSE – single factor, ANX – single factor, and DEP – single factor) were specified – each item was specified to load onto its specific factor. Secondly, an alternative model was specified: a first-order single factor model was specified – all the items of the abovementioned scales were loaded onto a single latent dimension. Models were sequentially specified and compared using the test differences in goodness-of-fit indices (Δχ^2^: *p* > 0.050; ΔCFI: >0.010; ΔRMSEA: >0.015). Model comparisons were based on typical interpretation guidelines: for example, a statistically significant chi-square difference (Δχ^2^; *p* < 0.050) and a ΔCFI greater than 0.010 suggest the absence of the bias (Meredith, 1993; Vandenberg and Lance, 2000; Cheung and Rensvold, 2002; Chen, 2007; Millsap, 2012; Brown, 2015).

#### Step 3

Latent factors were defined by using a partially disaggregated parcel approach in which latent constructs were defined by using parcels as indicators (Bandalos and Finney, 2001; Coffman and MacCallum, 2005; Little et al., 2013). More in detail, since four scales were unidimensional (FCV-19S, RSE, ANX, and the DEP), item parcels were created using the “item-to-construct balance strategy” (Bandalos and Finney, 2001; Little et al., 2002; Yang et al., 2010) – by inspecting factor loadings resulting from each measurement model (Little et al., 2002, 2013). However, since the UCLA-LS3 showed a hierarchical second-order structure, item parcels were created by using the “domain-representative strategy” (Kishton and Widaman, 1994; Graham et al., 2000; Little et al., 2002, 2013; Graham, 2004) – for each dimension, items were aggregated together. For each scale, at least a 3-item-parcel *per* latent variable were created – allowing each factor to be at least “just identified” – with factor loadings higher than |0.5| on the related construct (Hoyle, 2012; Little et al., 2013; Brown, 2015; Kline, 2016). Once item parcels were created, descriptive statistics were examined: an almost normal distribution was found for the large majority of parcels. Thus, the maximum likelihood (ML) estimator was used for each SEM described in the following step (“Step 4”) (Muthén and Muthén, 1998-2017; Hoyle, 2012; Kline, 2016). In addition, a 10,000 bootstrap resampling procedure was applied to each tested model (MacKinnon, 2012).

#### Step 4

**FIGURE 1:**
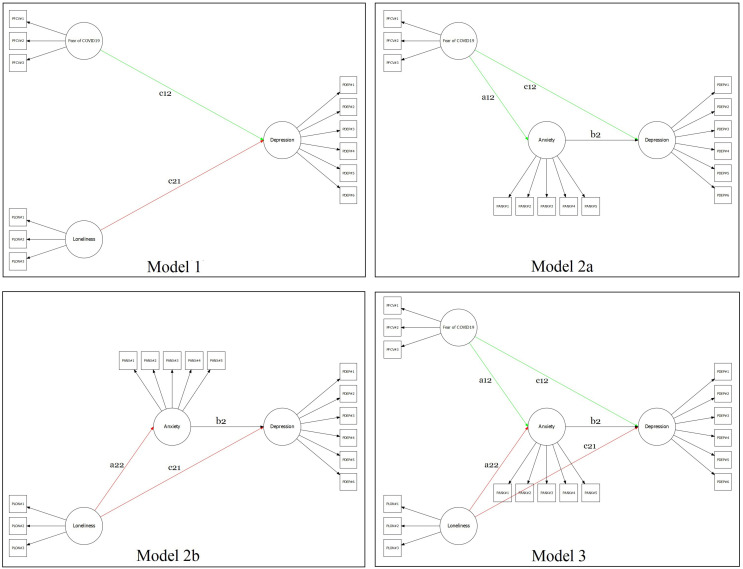
Graphical representation of the several mediation models tested.

**FIGURE 2:**
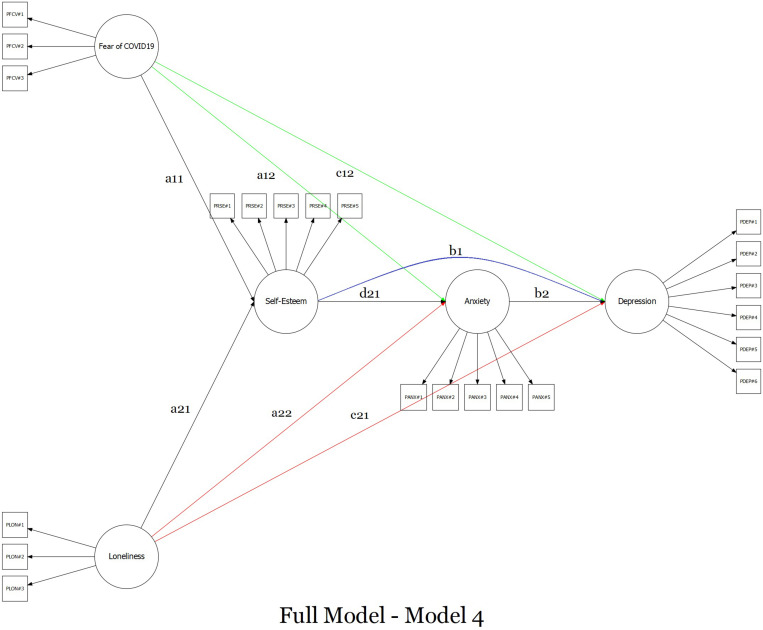
Graphical representation of the full sequential multiple mediation model with two-related different predictors.

The two-related separated predictors multiple mediation model was tested using a four-step approach (MacKinnon et al., 2007; Iacobucci, 2009; Rucker et al., 2011; MacKinnon, 2012). *Firstly*, a predictors-only model was specified: fear of COVID-19 (X_1_) and dispositional loneliness (X_2_) predict depressive symptomatology (Y) – Figure 1, Model 1. *Secondly*, a model was specified by excluding the effect of self-esteem (buffering variable) and dispositional loneliness: fear of COVID-19 (X_1_) predict depressive symptomatology (Y) through anxiety symptoms (M) – Figure 1, Model 2a. *Thirdly*, a parallel model was specified by excluding the effect of self-esteem (buffering variable) and fear of COVID-19: dispositional loneliness (X_2_) predicts depressive symptomatology (Y) through anxiety symptoms (M) – Figure 1, Model 2b. *Fourthly*, a semi-completed model was specified by only excluding the effect of self-esteem (buffering variable): fear of COVID-19 (X_1_) and dispositional loneliness (X_2_) predicts depressive symptomatology (Y) through anxiety symptoms (M) – Figure 1, Model 3. *Fifthly*, the final model was specified by including the buffering effect of self-esteem: fear of COVID-19 (X_1_) and dispositional loneliness (X_2_) predict depressive symptoms (Y) through self-esteem (M_1_) and anxiety symptoms (M_2_) – Model 4, Figure 2.

Each of the five models described in “Step 4” was evaluated using the abovementioned “goodness-of-fit” indices (χ^2^, RMSEA, CFI, SRMR) and their cutoffs values – and each tested model had to provide good fit indices (Frazier et al., 2004; Iacobucci, 2010). In addition, to avoid possible biases related to the scaling method (by default, the first factor loading of each latent variable was fixed to 1), an alternative model was specified by fixing the variance of each latent variable to unity (Gonzalez and Griffin, 2001). This procedure was repeated for each of the five models described above. Finally, all regression coefficients (β) reported in the text were unstandardized.

## Results

### Preliminary Analysis

The multivariate multiple regression analysis showed no statistically significant effects of external variables on psychological constructs. More in detail, controlling for other external variables, no statistically significant effect of (A) *Italian region of residence* was found on FCV-19S (β = 0.515, SE = 0.289, *z* = 1.786, *p* = 0.074), UCLA-LS3 (β = 0.290, SE = 0.518, *z* = 0.561, *p* = 0.575), RSE (β = −0.191, SE = 0.255, *z* = −0.751, *p* = 0.453), and DEP (β = 0.073, SE = 0.041, *z* = 1.800, *p* = 0.072). A negligible effect was found on ANX (β = 0.095, SE = 0.043, *z* = 2.207, *p* = 0.027). Moreover, controlling for other external variables, no statistically significant effect of the (B) *number of persons living with* was found on UCLA-LS3 (β = −0.377, SE = 0.236, *z* = −1.599, *p* = 0.110), RSE (β = 0.089, SE = 0.119, *z* = 0.747, *p* = 0.455), ANX (β = 0.029, SE = 0.020, *z* = 1.444, *p* = 0.149), and DEP (β = −0.017, SE = 0.019, *z* = −0.910, *p* = 0.363). A small effect was found on FCV-19S (β = 0.483, SE = 0.140, *z* = 3.443, *p* = 0.001). Moreover, controlling for other external variables, no statistically significant effect of (C) *confirmed positive COVID-19 diagnosis of the respondent* was found on FCV-19S (β = 0.556, SE = 0.544, *z* = 1.022, *p* = 0.307), UCLA-LS3 (β = −0.067, SE = 0.863, *z* = −0.077, *p* = 0.939), RSE (β = 0.508, SE = 0.394, *z* = 1.290, *p* = 0.197), ANX (β = 0.026, SE = 0.087, *z* = 0.302, *p* = 0.763), and DEP (β = −0.059, SE = 0.074, *z* = −0.800, *p* = 0.424). Finally, controlling for other external variables, no statistically significant effect of the presence of (D) *confirmed positive COVID-19 diagnosis of the respondents’ significant other* was found on FCV-19S (β = 0.100, SE = 0.268, *z* = 0.372, *p* = 0.710), UCLA-LS3 (β = 0.502, SE = 0.413, *z* = 1.217, *p* = 0.223), RSE (β = 0.086, SE = 0.205, *z* = 0.419, *p* = 0.675), ANX (β = −0.021, SE = 0.040, *z* = −0.511, *p* = 0.609), and DEP (β = −0.022, SE = 0.034, *z* = −0.667, *p* = 0.505).

In addition, correlation analyses suggested small-to-large associations between the variables involved in the multiple mediation model (Table 2).

**TABLE 2:** Mean, standard deviation, and correlations between observed variables.

		*M*	SD	1	2	3	4	5	6	7	8
1	FCV19S	19.63	5.678	−							
2	UCLA-LS3	43.34	9.353	0.161	−						
3	ISO	7.03	2.073	0.188	0.742	−					
4	REL. CON.	19.77	4.120	0.150	0.895	0.592	−				
5	T. LON	16.54	4.595	0.107	0.898	0.529	0.658	−			
6	RSE	29.44	4.533	–0.218	–0.532	–0.464	–0.494	–0.432	−		
7	ANX	1.05	0.832	0.717	0.296	0.303	0.268	0.226	–0.333	−	
8	DEP	1.19	0.755	0.419	0.578	0.564	0.517	0.459	–0.581	0.664	−

*Each correlation is statistically significant at p < 0.001; FCV, fear of COVID-19 scale; UCLA-LS3, UCLA loneliness scale; ISO, UCLA sense of isolation; REL. CON., UCLA sense of relational connectedness; T. LON, UCLA trait loneliness; RSE, Rosenberg self-esteem scale; ANX, SCL-90R anxiety scale; DEP, SCL-90R depression scale.*

### Structural Models

The FCV19S showed adequate goodness-of-fit indices: χ^2^ (14) = 88.338; *p* < 0.001; RMSEA = 0.067; 90%CI 0.054–0.080; *p*(RMSEA < 0.05) = 0.018, CFI = 0.996, SRMR = 0.038. Factor loadings of the items ranged from 0.705 (item#2) to 0.872 (item#5) (*mean* = 0.778; *SD* = 0.065).

The UCLA-LS3 showed adequate goodness-of-fit indices: χ^2^ (167) = 1261.908; *p* < 0.001; RMSEA = 0.074; 90%CI 0.070–0.078; *p*(RMSEA < 0.05) < 0.001, CFI = 0.985, SRMR = 0.059. Factor loadings of the first-order items ranged from 0.555 (item#7 – Relational connectedness) to 0.892 (item#14 – Relational connectedness) (*mean* = 0.719; *SD* = 0.157). Factor loadings of the second-order items ranged from 0.785 (Isolation) to 0.939 (Relational connectedness) (*mean* = 0.851; *SD* = 0.079).

Also the RSE revealed good results: χ^2^ (35) = 249.239; *p* < 0.001; RMSEA = 0.071; 90%CI 0.063–0.080; *p*(RMSEA < 0.05) < 0.001, CFI = 0.990, SRMR = 0.052. Factor loadings of the items ranged from 0.541 (item#4) to 0.817 (item#2) (*mean* = 0.704; *SD* = 0.105).

Even the ANX showed good fit indices: χ^2^ (35) = 208.462; *p* < 0.001; RMSEA = 0.064; 90%CI 0.056–0.073; *p*(RMSEA < 0.05) = 0.003, CFI = 0.997, SRMR = 0.036. Factor loadings of the items ranged from 0.768 (item#2) to 0.887 (item#3) (*mean* = 0.830; *SD* = 0.043).

Finally, also the DEP revealed good fit indices: χ^2^ (65) = 310.064; *p* < 0.001; RMSEA = 0.056; 90%CI 0.050–0.062; *p*(RMSEA < 0.05) = 0.053, CFI = 0.994, SRMR = 0.046. Factor loadings of the items ranged from 0.448 (item#1) to 0.896 (item#8) (*mean* = 0.724; *SD* = 0.110).

### Harman’s Single-Factor Test

The Harman’s single-factor test showed the absence of the “common method bias.” Indeed, the CFA with seven correlated factors (FCV19 – single factor, UCLA-LS3– three factors, RSE – single factor, ANX – single factor, and DEP – single factor) provided good fit indices [χ^2^ (1689) = 8434.991; *p* < 0.001; RMSEA = 0.058; 90%CI 0.056–0.059; *p*(RMSEA < 0.05) < 0.001, CFI = 0.983, SRMR = 0.060]. Contrarily, the CFA with a single latent factor provided poor fit indices [χ^2^ (1710) = 54429.649; *p* < 0.001; RMSEA = 0.160; 90%CI 0.159–0.162; *p*(RMSEA < 0.05) < 0.001, CFI = 0.866, SRMR = 0.147]. Model comparison suggested the absence of the “common method bias”: Δχ^2^ (21) = 45995, *p* < 0.001; |ΔRMSEA| = 0.103, and |ΔCFI| = 0.117.

### Multiple Mediation Model

#### Model 1

The first model (Figure 1, model 1) provided adequate goodness-of-fit indices: χ^2^ (51) = 377.938; *p* < 0.001; RMSEA = 0.073; 90%CI 0.066–0.080; *p*(RMSEA < 0.05) < 0.001, CFI = 0.964, SRMR = 0.043 (Table 3). The fear of COVID-19 (X_1_) was positively associated with depressive symptomatology (Y): β = 0.537 (SE = 0.047) [95%CI: 0.452; 0.632], *z* = 11.551, *p* < 0.001. At the same time, the dispositional loneliness (X_2_) was positively associated with depressive symptomatology (Y): β = 0.932 (SE = 0.060) [95%CI: 0.822; 1.057], *z* = 15.484, *p* < 0.001. Moreover, fear of COVID-19 and loneliness were statistically significantly associated: β = 0.199 (SE = 0.035) [95%CI: 0.129; 0.267], *z* = 5.601, *p* < 0.001.

**TABLE 3:** Parcel descriptive statistics and standardized factor loadings (λ).

	Descriptive statistics				Model 1	Model 2a	Model 2b	Model 3	Model 4
	*M*	SD	SK	K	λ	λ	λ	λ	λ
**FCV-19Ss (X_1_)**									
pFCV#1	2.905	0.984	0.112	–0.636	0.869	0.876	−	0.876	0.876
pFCV#2	3.015	0.809	0.197	–0.084	0.839	0.839	−	0.839	0.839
pFCV#3	2.595	0.868	0.290	–0.352	0.896	0.890	−	0.890	0.890
**UCLA-LS3 (X_2_)**									
pFCV#1	2.344	0.691	–0.028	–0.548	0.734	−	0.736	0.736	0.735
pFCV#2	2.471	0.515	0.046	–0.205	0.828	−	0.828	0.838	0.830
pFCV#3	1.838	0.511	0.540	0.216	0.757	−	0.757	0.757	0.755
**RSE (M_1_)**									
pRSE#1	2.901	0.562	–0.077	0.191	−	−	−	−	0.790
pRSE#2	3.011	0.449	–0.727	3.481	−	−	−	−	0.725
pRSE#3	3.059	0.580	–0.362	0.263	−	−	−	−	0.807
pRSE#4	3.035	0.492	–0.504	1.815	−	−	−	−	0.766
pRSE#5	2.714	0.662	–0.048	–0.172	−	−	−	−	0.777
**ANX (M_2_)**									
pANX#1	0.617	0.808	1.470	1.820	−	0.836	0.841	0.836	0.836
pANX#2	1.148	0.965	0.787	0.000	−	0.896	0.894	0.897	0.897
pANX#3	0.769	0.908	1.228	0.931	−	0.856	0.861	0.856	0.856
pANX#4	0.987	0.976	1.021	0.417	−	0.882	0.880	0.882	0.881
pANX#5	1.716	1.030	0.193	–0.648	−	0.821	0.817	0.821	0.821
**DEP (Y)**									
pDEP#1	1.569	0.946	0.398	–0.391	0.782	0.783	0.781	0.780	0.777
pDEP#2	1.379	0.970	0.513	–0.379	0.783	0.770	0.777	0.777	0.783
pDEP#3	1.424	0.955	0.489	–0.252	0.764	0.760	0.760	0.760	0.760
pDEP#4	1.150	0.934	0.735	0.046	0.835	0.843	0.834	0.834	0.835
pDEP#5	0.761	0.703	0.980	0.991	0.789	0.803	0.798	0.797	0.794
pDEP#6	0.985	0.893	0.997	0.505	0.844	0.835	0.845	0.846	0.846

*Each item-parcel factor loading (λ) is statistically significant at p < 0.001; p(…), item parcel; FCV-19S, fear of COVID-19 scale; UCLA-LS3, UCLA loneliness scale; RSE, Rosenberg self-esteem scale; ANX, SCL-90R anxiety scale; DEP, SCL-90R depression scale.*

#### Model 2a

The second model (Figure 1, model 2a) provided adequate goodness-of-fit indices: χ^2^ (74) = 505.982; *p* < 0.001; RMSEA = 0.070; 90%CI 0.064–0.076; *p*(RMSEA < 0.05) < 0.001, CFI = 0.968, SRMR = 0.039 (Table 3). The fear of COVID-19 (X_1_) was positively associated with anxiety symptomatology (M): β = 1.257 (SE = 0.064) [95%CI: 1.140; 1.390], *z* = 19.566, *p* < 0.001. Moreover, anxiety symptomatology (M) predicted depressive symptoms (Y): β = 0.827 (SE = 0.054) [95%CI: 0.724; 0.937], *z* = 15.321, *p* < 0.001. Also, fear of COVID-19 was negatively associated with depressive symptomatology: β = −0.338 (SE = 0.069) [95%CI: −0.476; −0.205], *z* = −4.865, *p* < 0.001. Furthermore, the total indirect effect was statistically significant [β = 1.039 (SE = 0.072) [95%CI: 0.908; 1.188], *z* = 14.372, *p* < 0.001] as well as the total model effect [β = 0.701 (SE = 0.058) [95%CI: 0.590; 0.821], *z* = 11.986, *p* < 0.001] – thus suggesting a partially mediated path.

#### Model 2b

The third model (Figure 1, model 2b) provided adequate goodness-of-fit indices: χ^2^ (74) = 583.259; *p* < 0.001; RMSEA = 0.076; 90%CI 0.070–0.082; *p*(RMSEA < 0.05) < 0.001, CFI = 0.958, SRMR = 0.043 (Table 3). Dispositional loneliness (X_2_) was positively associated with anxiety symptomatology (M): β = 0.366 (SE = 0.038) [95%CI: 0.293; 0.442], *z* = 9.631, *p* < 0.001. Moreover, anxiety symptomatology (M) predicted depressive symptomatology (Y): β = 0.988 (SE = 0.063) [95%CI: 0.874; 1.121], *z* = 15.752, *p* < 0.001. In this case, dispositional loneliness was positively associated with depressive symptomatology: β = 0.931 (SE = 0.066) [95%CI: 0.806; 1.065], *z* = 14.025, *p* < 0.001. The total indirect effect was statistically significant [β = 0.361 (SE = 0.042) [95%CI: 0.285; 0.449], *z* = 8.660, *p* < 0.001] as well as the total model effect [β = 1.292 (SE = 0.080) [95%CI: 1.147; 1.459], *z* = 16.074, *p* < 0.001] – thus suggesting, a partially meditated model.

#### Model 3

The fourth model (Figure 1, model 3) provided adequate goodness-of-fit indices: χ^2^ (113) = 703.306; *p* < 0.001; RMSEA = 0.066; 90%CI 0.061–0.071; *p*(RMSEA < 0.05) < 0.001, CFI = 0.962, SRMR = 0.043 (Table 3). As shown for “Model 1,” fear of COVID-19 (X_1_) and dispositional loneliness (X_2_) were positively associated: β = 0.199 (SE = 0.036) [95%CI: 0.128; 0.270], *z* = 5.523, *p* < 0.001. Fear of COVID-19 (X_1_) was also positively associated with anxiety symptomatology (M): β = 1.256 (SE = 0.064) [95%CI: 1.136; 1.389], *z* = 19.713, *p* < 0.001. At the same time, dispositional loneliness (X_2_) was positively associated with anxiety symptomatology (M): β = 0.330 (SE = 0.040) [95%CI: 0.251; 0.410], *z* = 8.179, *p* < 0.001. Moreover, anxiety symptomatology (M) predicted depressive symptomatology (Y): β = 0.722 (SE = 0.060) [95%CI: 0.661; 0.896], *z* = 12.938, *p* < 0.001. Also, as shown in “Model 2a” fear of COVID-19 was negatively associated with depressive symptomatology [β = −0.288 (SE = 0.079) [95%CI: −0.451; −0.138], *z* = −3.639, *p* < 0.001] and, as for “Model 2b,” dispositional loneliness was positively associated with depressive symptomatology: β = 0.924 (SE = 0.067) [95%CI: 0.801; 1.064], *z* = 13.852, *p* < 0.001.

The first total indirect effect (fear of COVID-19 → anxiety symptomatology → depressive symptomatology) was statistically significant [β = 0.970 (SE = 0.082) [95%CI: 0.822; 1.145], *z* = 11.785, *p* < 0.001] as well as the total model effect [β = 0.682 (SE = 0.060) [95%CI: 0.579; 0.806], *z* = 11.306, *p* < 0.001] – thus suggesting a partially mediated model. In addition, the second total indirect effect (dispositional loneliness → anxiety symptomatology → depressive symptomatology) was statistically significant [β = 0.255 (SE = 0.034) [95%CI: 0.191; 0.326], *z* = 7.427, *p* < 0.001] as well as the total model effect [β = 1.179 (SE = 0.078) [95%CI: 1.187; 1.714], *z* = 15.102, *p* < 0.001] – thus suggesting a partially mediated model.

#### Model 4

The final model (Figure 2) provided satisfying goodness-of-fit indices: χ^2^ (199) = 918.943; *p* < 0.001; RMSEA = 0.055; 90%CI 0.051–0.059; *p*(RMSEA < 0.05) = 0.012, CFI = 0.962, SRMR = 0.039 (Table 3). As shown for “Model 1,” fear of COVID-19 (X_1_) and dispositional loneliness (X_2_) were positively associated: β = 0.199 (SE = 0.036) [95%CI: 0.126; 0.269], *z* = 5.484, *p* < 0.001. According to the ABH, fear of COVID-19 (X_1_) was negatively associated with self-esteem (M_1_): β = −0.160 (SE = 0.040) [95%CI: −0.237; −0.082], *z* = −4.015, *p* < 0.001, and self-esteem – in turn – negatively predicted anxiety symptomatology (M_2_): β = −0.127 (SE = 0.045) [95%CI: −0.216; −0.039], *z* = −2.797, *p* = 0.005 – thus revealing the buffering effect of self-esteem. Finally, anxiety symptomatology (M_2_) positively predicted depressive symptomatology (Y): β = 0.769 (SE = 0.060) [95%CI: 0.657; 0.894], *z* = 12.775, *p* < 0.001. In addition, in line with the ABH, self-esteem (M_1_) was negatively associated with depressive symptomatology (Y): β = −0.371 (SE = 0.052) [95%CI: −0.474; −0.269], *z* = −7.095, *p* < 0.001 – further suggesting the buffering effect of self-esteem. Furthermore, fear of COVID-19 (X_1_) was positively associated with anxiety symptomatology (M_2_) [β = 1.245 (SE = 0.065) [95%CI: 1.128; 1.380], *z* = 19.283, *p* < 0.001] and in line with “Model 2a” and “Model 3” fear of COVID-19 (X_1_) was negatively associated with depressive symptomatology (Y) [β = −0.309 (SE = 0.079) [95%CI: −0.471; −0.159], *z* = −3.924, *p* < 0.001].

At the same time, according to the ABH, dispositional loneliness (X_2_) was negatively associated with self-esteem (M_1_): β = −0.798 (SE = 0.055) [95%CI: −0.913; −0.695], *z* = −14.403, *p* < 0.001 – revealing the buffering effect of self-esteem. Furthermore, dispositional loneliness (X_2_) was positively associated with anxiety symptomatology (M_2_) [β = 0.231 (SE = 0.055) [95%CI: 0.125; 0.341], *z* = 4.211, *p* < 0.001] and, in line with “Model 2b” and “Model 3,” also positively associated with depressive symptomatology (Y) [β = 0.703 (SE = 0.072) [95%CI: 0.570; 0.854], *z* = 9.700, *p* < 0.001].

The first total indirect effect (fear of COVID-19 → self-esteem → anxiety symptomatology → depressive symptomatology) was statistically significant [β = 0.016 (SE = 0.007) [95%CI: 0.004; 0.030], *z* = 2.324, *p* = 0.020] as well as the total model effect [β = 0.724 (SE = 0.064) [95%CI: 0.604; 0.858], *z* = 11.252, *p* < 0.001] – suggesting a partially mediated model that highlighted the buffering effect of self-esteem.

In addition, the second total indirect effect (dispositional loneliness → self-esteem → anxiety symptomatology → depressive symptomatology) was statistically significant [β = 0.078 (SE = 0.030) [95%CI: 0.023; 0.140], *z* = 2.634, *p* = 0.008] as well as the total model effect [β = 1.154 (SE = 0.083) [95%CI: 1.008; 1.332], *z* = 13.967, *p* < 0.001] – thus suggesting a partially mediated model with the buffering effect of self-esteem (Table 4).

**TABLE 4:** Summary of parameter estimates (beta) with 95% confidence intervals for key pathways tested full model, Model 4 – Figure 2.

Path		B	β (SE)	95% CI [L–U]	*z-value*	*p*-value	*R*^2^
Fear of COVID-19 (X_1_) → self-esteem (M_1_)	(a11)	–0.122	−0.160(0.040)	[−0.237; −0.082]	–4.015	*p* < 0.001	
Loneliness (X_2_) → self-esteem (M_1_)	(a21)	–0.610	−0.798(0.055)	[−0.913; −0.695]	–14.403	*p* < 0.001	0.416
Self-esteem (M_1_) → anxiety (M_2_)	(d21)	–0.098	−0.127(0.045)	[−0.216; −0.039]	–2.797	*p* = 0.005	0.655
Anxiety (M_2_) → depression (Y)	(b2)	0.633	0.769 (0.060)	[0.657; 0.894]	12.775	*p* < 0.001	0.766
Fear of COVID-19 (X_1_) → anxiety (M_2_)	(a12)	0.732	1.245 (0.065)	[1.128; 1.380]	19.283	*p* < 0.001	
Fear of COVID-19 (X_1_) → depression (Y)	(c11)	–0.149	−0.309(0.079)	[−0.471; −0.159]	–3.924	*p* < 0.001	
Loneliness (X_2_) → anxiety (M_2_)	(a22)	0.136	0.231 (0.052)	[0.125; 0.341]	4.211	*p* < 0.001	
Loneliness (X_2_) → depression (Y)	(c21)	0.340	0.703 (0.072)	[0.570; 0.854]	9.700	*p* < 0.001	
Self-esteem (M_1_) → depression (Y)	(b1)	–0.235	−0.371(0.052)	[−0.474; −0.269]	–7.095	*p* < 0.001	
Indirect effect of X_1_ on Y via M_1_	(a11*b1)	0.029	0.059 (0.017)	[0.029; 0.094]	3.495	*p* < 0.001	
Indirect effect of X_1_ on Y via M_2_	(a12*b2)	0.463	0.958 (0.082)	[0.813; 1.134]	11.714	*p* < 0.001	
Indirect effect of X_2_ on Y via M_1_	(a21*b1)	0.143	0.296 (0.044)	[0.214; 0.386]	6.744	*p* < 0.001	
Indirect effect of X_2_ on Y via M_2_	(a22*b2)	0.086	0.178 (0.043)	[0.097; 0.268]	4.098	*p* < 0.001	
Indirect effect of X_1_ on Y via M_1_ and M_2_	(a11*d21*b2)	0.008	0.016 (0.007)	[0.004; 0.030]	2.324	*p* = 0.020	
Indirect effect of X_2_ on Y via M_1_ and M_2_	(a21*d21*b2)	0.038	0.078 (0.030)	[0.023; 0.140]	2.634	*p* = 0.008	
Total effect X_1_ on Y		0.350	0.724 (0.064)	[0.604.; 0.858]	11.252	*p* < 0.001	
Total effect X_2_ on Y		0.561	1.154 (0.083)	[1.008; 1.332]	13.967	*p* < 0.001	

*B, standardized beta; β, unstandardized beta; 95%CI, 95% confidence intervals for the unstandardized beta. SE, standard error.*

## Discussion

Recently, the potential negative impact that the adverse psychological consequences of COVID-19 further had on the disease itself have been highlighted in the literature (Center for Disease Control and Prevention, 2020; Lima et al., 2020; Parola, 2020; Parola et al., 2020; Thakur and Jain, 2020; Wind et al., 2020). Indeed, the advent of COVID-19 generated intense fear and anxiety about contagion, disease, and thoughts of death in the general population. At the same time, the sense of isolation was amplified by dispositional loneliness during the COVID-19 lockdown, with a consequent increase of anxiety symptoms. Therefore, both a fear of COVID-19 and dispositional loneliness represent major risk factors for the development of symptoms of anxiety and following symptoms of depression.

This study highlighted the buffering-effect of self-esteem on the relationships between negative psychological constructs, such as a fear of COVID-19 and dispositional loneliness feelings (predictors), and their consequent adverse psychological correlates – anxiety and depression (outcomes) during the COVID-19 pandemic.

In line with the scientific literature showing that (prolonged) fear can lead to depression (Bowman, 2001), this study revealed that a fear of COVID-19 and loneliness might lead to depressive symptoms (Santini et al., 2020; Thakur and Jain, 2020). Indeed, the first model that has been tested (Model 1 – predictors only) showed a positive relationship between a fear of COVID-19 and depressive symptomatology, with higher fear predicting higher depressive symptomatology. Indeed, when controlling for loneliness, an increase of 1 point in fear of COVID-19 was associated with an increase of 0.537 points in depression. At the same time, loneliness was positively associated with depressive symptoms: an increase in 1 point in dispositional loneliness was associated with an increase of 0.932 points in depression. These results suggest that a prolonged state of fear and dispositional feelings of loneliness might lead to the development of adverse psychological symptoms – thus representing major risk factors for the onset of symptoms of depression.

However, when controlling for anxiety activation (Model 3), fear (of COVID-19) and depression showed a negative association, probably due to the different nature of these emotional states. Indeed, on one hand, fear represents an activating emotion prompting the organism to react with the well-known *“fight or flight”* response. On the other hand, depression is characterized by a generalized de-activation, reflected in slowed-down behavior and thinking as well as flattened affectivity and pleasure (Beck, 1979; Harper et al., 2020). At the same time, fear was positively strongly associated with anxiety symptoms (Barlow, 2002; Harding et al., 2008), which might lead to depression (Bowman, 2001) – thus suggesting a partially mediated model starting from fear up to depression through anxiety.

Simultaneously – when controlling for anxiety – dispositional loneliness was positively associated with depressive symptoms, further highlighting the existence of a strong relationship between these two constructs (Cacioppo et al., 2014; Santini et al., 2020). Also, dispositional loneliness was positively associated with anxiety symptoms leading to depression (Thompson et al., 2005; Starr and Davila, 2012) – suggesting, a partially mediated model (Model 3).

However, in line with the hypotheses, the final model (Model 4) highlighted the buffering role of self-esteem: despite positive associations held between fear of COVID-19, dispositional loneliness, and anxiety, the effect of self-esteem slowed down these negative adverse paths. Indeed – in line with the ABH and the TMT (Greenberg et al., 1986, 1992) – self-esteem had negative relationships with all the other psychological constructs (negative β values) due to its buffering effect hampering the relationships between adverse psychological variables. A partial mediation model was, therefore, suggested given that the relationship between fear of COVID-19, dispositional loneliness, anxiety, and depression held even when their paths were buffered by self-esteem.

Summarizing, results showed that self-esteem had a buffer effect protecting against anxiety symptoms triggered by a fear of COVID-19 and dispositional loneliness. Thus, these findings confirmed the validity of the ABH in the context of the COVID-19 pandemic.

Results also highlighted that both a fear of COVID-19 and dispositional loneliness were able to trigger unbearable feelings of anxiety that, in turn, were strongly linked to depressive symptomatology.

The strict interconnection between self-esteem and loneliness was probably due to the fact that loneliness is often related to negative self-evaluations, and feelings of being worthless, inferior, or unlovable (Heinrich and Gullone, 2006). Previous studies suggested that self-esteem may impact on loneliness as a reinforcer or a buffer, as instances of influencing the relational competences (Olmstead et al., 1991; Brage and Meredith, 1994; Heinrich and Gullone, 2006).

These results are in line with previous scientific literature highlighting that self-esteem can be a mediator in the relationship between loneliness, anxiety, and depression (Brage and Meredith, 1994; Heinrich and Gullone, 2006; Çivitci and Çivitci, 2009).

Regarding the clinical implications of this study, its findings suggest a possible intervention strategy to provide psychological support to people suffering from the emotional consequences of COVID-19 and other COVID-19-related issues in order to alleviate the psychological outbreak of the pandemic. Indeed, according to the ABH, if self-esteem provides protection against stressors, such sources of stress should increase the need for self-esteem to relieve psychological burden (Harmon-Jones et al., 1997). Consequently, increased self-esteem should function as a buffer toward anxiety, reducing the adverse psychological issues in response to threats or stressors. Thus, psychological interventions targeting self-esteem can represent an effective strategy to attenuate the distressing psychological responses to COVID-19 fear and dispositional feelings of loneliness – particularly among populations most susceptible and vulnerable to the negative psychological effects of the COVID-19 pandemic, including people with psychiatric disorders, those at risk of domestic violence, elderly people, and health-care practitioners (Lai et al., 2020; Armitage and Nellums, 2020; Yao et al., 2020).

Moreover, given that loneliness derives from the perceived discrepancy between the actual and desired quality of relationships (Peplau and Perlman, 1982), these results highlight the importance of perceived social support and positive relationships for people (Ratti et al., 2017; Panzeri et al., 2019; Duan and Zhu, 2020). Individuals should, therefore, be guided and educated in strengthening their relationships and social support resources when physical contact is not possible (i.e., quarantine, hospitalization) by adopting tele-communication tools, such as smartphones. In line with the debated internet-paradox, proper technology use should be promoted to prevent distressing feelings (Moody, 2001; Enez Darcin et al., 2016; Király et al., 2020).

Some noteworthy limitations of this study need to be acknowledged. Due to the observational/correlational nature of the research design, it was not possible to establish a causal relationship among variables, but only predictive relationships – still in line with the study purpose (Fiedler et al., 2011). Moreover, the self-report nature of the online survey may convey intrinsic biases related to social desirability and other well-known issues (Vidotto et al., 2018). Other limitations of this study were the prevalence of females in the sample and that the fact that geographical areas in Italy were not equally represented – although preliminary analyses showed no associated effects. Likewise, no differences emerged from sociodemographic characteristics, but future studies should deepen their possible role as protective/risk factors (i.e., presence vs. absence of social support) (Mannarini et al., 2017a). In addition, multi-group analyses assessing tested models across sex (males vs. females) were not performed. However, due to the small male sample size, multi-group mediation analyses would not be able to provide an accurate estimation of model parameters (Hoyle, 2012; Kline, 2016). Future studies should, therefore, further test potential effects of sex on the suggested models. Moreover, all participants were Italian and possible effects of cross-cultural differences cannot be considered. Even though the ABH was successfully replicated in various countries as well as in different contexts (Pyszczynski et al., 2004), future studies specifically examining the impact of COVID-19 on people’s lives should compare these results among different countries thus increasing the generalizability of these findings.

Finally, a mediation model was preferred to a moderation one for both theoretical and statistical reasons. Indeed, from a theoretical perspective a mediation-based approach is closer and more related to the original ABH and the TMT (Greenberg et al., 1986, 1992), conceptualizing self-esteem as an intermediating buffer between life-threatening stressors and anxiety (Pyszczynski et al., 2004). In fact, self-esteem not only is able to influence individuals’ levels of anxiety and depression, but it is itself influenced by negative psychological experiences – such as fear and loneliness – activating negative cognitions and emotions that significantly affect the idea of oneself (Greenberg et al., 1986; Heinrich and Gullone, 2006; Sowislo and Orth, 2013). Research show that fear can threaten self-evaluation (Greenberg et al., 1986), and that people experiencing higher feelings of loneliness also have a worse self-evaluation (Heinrich and Gullone, 2006). More in detail, negative experiences can activate both negative cognitions and emotions that significantly affect the idea of oneself (i.e., “I am a failure”, “I am worthless”) (Beck, 1979) – thus, leaving scars in the self-concept, as well as persistently threatening and reducing self-esteem and self-efficacy (Mannarini, 2010; Sowislo and Orth, 2013). Thus, a moderation approach would not suit the theoretical background of this study, and would not allow us to properly take into account the complexity of relationships among the considered psychological constructs. Regarding the strengths of the present study, it relies on a well-grounded theoretical basis supported by several experimental and longitudinal studies (Greenberg et al., 1992; Brage and Meredith, 1994; Pyszczynski et al., 2004; Heinrich and Gullone, 2006). A wide sample of individuals from the general population was analyzed with strong statistical methodologies (Iacobucci et al., 2007; MacKinnon, 2012; MacKinnon et al., 2012, 2013) providing good results (McDonald and Ho, 2002; Hayes, 2009; Iacobucci, 2010; Preacher, 2015). Moreover, the hypothesized models resulted in having a good fit, even if other solutions would have been possible but with lower fit indexes.

Given that individuals faced similar problems during past epidemics, findings from this study could also be generalized and applied to support people still coping with the negative consequences that previous disease outbreaks had on their mood (i.e., Ebola, SARS, MERS, and tuberculosis) (Brown and Lees-Haley, 1992; Betancourt et al., 2016; Huremović, 2019; Chew et al., 2020). In a broader sense, these results could be extended to relieve the psychological burden of dysfunctional psychological reactions in response to physical and/or psychological illnesses (Rossi Ferrario and Panzeri, 2020).

Overall, this study contributes to the current debate about the psychological implications of the COVID-19 pandemic, a prolonged and distressing situation triggering or worsening psychological issues. These findings may also be useful to help clinicians develop efficient and tailored interventions for increasing individuals’ mental health – with particular attention to the more fragile categories, such as young people and elderly people (Parola and Donsì, 2018, 2019; Balestroni et al., 2020).

Although, a considerable number of individuals may avoid seeking professional psychological help (Rossi and Mannarini, 2019) due to the associated stigma (Mannarini et al., 2017b, 2018, 2020; Faccio et al., 2019; Mannarini and Rossi, 2019) or because of defensive denial reactions toward their psychological difficulties (Sareen et al., 2007; Rossi Ferrario et al., 2019), thus choosing to manage the psychological issues on their own (Wilson and Deane, 2012).

Future research will provide further insight about the evolution over time of the psychological issues related to COVID-19. Future studies might examine the role of social support as well as the changes in the dynamics of social and family relationships (Mannarini et al., 2013, 2017a; Balottin et al., 2017).

Still, the role of other psychological constructs that may act as protective or risk factors, such as anger, post-traumatic symptoms, hopelessness, and denial should be further explored in future research in order to find effective treatment strategies to adopt in order to deal with consequences of both the COVID-19 and future pandemics.

## Conclusion

The present research offers further support for the anxiety-buffer role of self-esteem, confirming TMT to be a well-grounded theoretical framework offering interesting and useful clinical insights in the context of the COVID-19 pandemic. Targeted psychological interventions should be implemented to properly support individuals suffering from COVID-19-related issues in order to minimize the psychological burden of the disease whilst promoting adaptation and positive psychological health outcomes.

## Data Availability Statement

The raw data supporting the conclusions of this article will be made available by the authors, on reasonable requests.

## Ethics Statement

The studies involving human participants were reviewed and approved by the Ethic Committee of the University of Padua. The patients/participants provided their written informed consent to participate in this study.

## Author Contributions

AR conceived the study, performed the statistical analyses, and wrote the first draft. AP wrote a large part of the first draft and collected the data. GP helped with data collection and wrote part of the first draft. GM, GC, and SM provided important intellectual revisions. All authors contributed to the article and approved the submitted version.

## Conflict of Interest

The authors declare that the research was conducted in the absence of any commercial or financial relationships that could be construed as a potential conflict of interest.
